# “It’s the modern way of doing things”: a qualitative study of user-experience, acceptability, and content validity of a digital mental health screener for refugees

**DOI:** 10.3389/fpsyg.2026.1902434

**Published:** 2026-09-11

**Authors:** Jennifer Meurling, Anna Bjärtå, Youstina Demetry, Anahita Geranmayeh, Gerhard Andersson, Elisabet Rondung

**Affiliations:** 1 Department of Psychology and Social Work, Mid Sweden University, Östersund, Sweden; 2 Department of Clinical Neuroscience, Centre for Psychiatry Research, Karolinska Institute & Stockholm Health Care Services, Stockholm, Sweden; 3 Department of Behavioral Sciences and Learning, Linköping University, Linköping, Sweden; 4 HEI-Lab: Digital Human-Environment Interaction Labs, Lusófona University, Lisbon, Portugal; 5 Department of Health, Education and Technology, Luleå University of Technology, Luleå, Sweden

**Keywords:** acceptability, content validity, digital mental health, e-health, qualitative research, refugees, validation, screening

## Abstract

**Background:**

Digital mental health tools hold potential to bridge barriers to services, and efficient screening could facilitate access and assessment. An internet-based tiered assessment procedure (i-TAP) screening for common psychiatric symptoms among refugees has been developed and validated in previous work. Further validation requires investigation of acceptability and content validity, which in turn can inform potential implementation.

**Aim:**

This study aimed to explore the user experience with the i-TAP among individuals with a refugee background in Sweden, with a particular focus on acceptability and content validity.

**Methods:**

In conjunction with a psychometric validation study of the i-TAP, semi-structured interviews were conducted with 71 adult participants with a refugee background living in Sweden. Interviews were conducted in Arabic, Dari, Farsi and Swedish, and analysed using reflexive thematic analysis.

**Results:**

Four themes were generated. Two themes addressed the content and usability of the i-TAP: *Capturing what matters – but not everything that matters and A user-friendly and accessible tool - provided sufficient (health) literacy*. A third theme: *Digital mental health screening could facilitate care, but can not replace a care visit*, highlighted perspectives on prospective implementation. The fourth theme, Sharing can be difficult, but opens the door to support, reflected emotional and cultural aspects of addressing mental health issues.

**Conclusion:**

Participants mainly found the i-TAP to be relevant and easy to use, and the digital format familiar. Items were comprehensible and relevant topics were covered, supporting content validity. Concurrent and prospective acceptability was high; however, with the prerequisite that screening must lead to action. The results highlighted the importance of trust and language when addressing mental health. We suggest developments to increase usability and comprehensiveness, and discuss considerations important for implementation.

## Introduction

Refugees face numerous traumatic and stressful events, and endure ongoing stress in forms of discrimination, language barriers, long asylum processes, short-term residence permits, unemployment and more, all of which are associated with poor mental health (Bogic et al., 2015; Porter and Haslam, 2005; Malm et al., 2020; Borho et al., 2020). Refugees are continuously found to have higher levels of psychiatric symptoms and disorders compared to the general population (Blackmore et al., 2020; Fazel et al., 2005; Patanè et al., 2022).

Despite the high prevalence of mental health problems, refugees are often underrepresented in mental health services (Derr, 2016; Satinsky et al., 2019; Due et al., 2020). Barriers to care include language difficulties, fear of stigma, difficulties navigating the health care system, and lack of trust (Satinsky et al., 2019; Kiselev et al., 2020; Mabil-Atem et al., 2024), as well as economical concerns, practical and logistical issues (Byrow et al., 2020; Mabil-Atem et al., 2024). From a care provider perspective, additional barriers such as communication problems and limited contact with refugee populations have been identified (Dumke et al., 2023). Taken together, these findings highlight barriers on both the supply and demand sides of care for individuals with a refugee background.

Digital mental health approaches hold potential to bridge this treatment gap by complementing established forms of care with flexible and tailored tools (Ng et al., 2025). Digital screening could facilitate access to, and guide efficient delivery of mental health services (Smith et al., 2023), as screening can serve as the first step toward suitable interventions. A digital screening format offers flexibility and can reduce costs as well as travel- and time demands on the individual (Whitehead et al., 2023; Liem et al., 2021). Furthermore, it facilitates delivery in different languages.

Against these potential advantages, and with the scalable and cost-effective nature of digital health tools in mind, we have designed and evaluated an internet-based tiered assessment procedure (herein referred to as the i-TAP) screening for common mental health problems among individuals with a refugee background. The i-TAP is tailored for populations with refugee experiences residing in Sweden and designed to identify clinically relevant symptoms of depression, anxiety, PTSD, and insomnia, using validated self-assessment scales. The procedure is adaptive to the respondent’s answers. Thus, an individual reporting few or mild symptoms will answer fewer questions than an individual reporting stronger and/or comorbid symptoms, see Meurling et al. (2026). We have evaluated the i-TAP psychometrically and against gold standard structured clinical interviews in Arabic, Dari, Farsi, Swedish and English. It has demonstrated high diagnostic accuracy and efficiency, supporting the criterion validity of the i-TAP (Meurling et al., 2023, 2026). Furthermore, a vast majority of participants rated a positive attitude toward digital mental health screening, the user experience as positive, the method as straightforward and the questions easy to understand, suggesting an acceptable and comprehensible tool (Meurling et al., 2026).

In addition to psychometric evaluation and criterion validity, it is highly relevant to investigate the content validity and acceptability of a measurement and procedure (Sireci, 1998; Skivington et al., 2021; Terwee et al., 2018). In digital health research, acceptability is a central concept (van Gemert-Pijnen et al., 2011), and previous studies involving refugee populations have identified specific barriers such as concerns about confidentiality and privacy, and limited access to the internet and devices (Mabil-Atem et al., 2024), that may impede acceptability and engagement. With future prospects in mind, successful implementation requires a procedure that works, is acceptable, accessible, and that measures what patients see as important (Perski and Short, 2021; Smith et al., 2023).

Central for this study was to investigate the content validity, usability and acceptability of the i-TAP from the perspective of end users. Content validity was conceptualized in accordance with the COnsensus-based Standards for the selection of health Measurement INstruments (COSMIN) guidelines (Terwee et al., 2018), encompassing the participants’ perception of the relevance, comprehensiveness, and comprehensibility of the i-TAP as a mental health screener for refugees. Acceptability was broadly defined as a subjective evaluation including how one thinks and feels about an intervention, usage intentions, user engagement, and satisfaction after engaging with the intervention (Sekhon et al., 2017; Nadal et al., 2020). Usability was addressed separately, referring specifically to the perceived ease of use (Shackel, 2009).

This study was conducted as part of a larger project with the aim to develop and evaluate a digital screener for mental health problems among refugees. The overall purpose of this qualitative component was to further explore user experience, focusing on the acceptability and perceived content validity of the i-TAP among individuals with a refugee background in Sweden. Additionally, we aimed to identify areas for development and factors critical for potential implementation from the perspective of the end users.

## Materials and methods

This interview study formed part of the SAHA project, a research initiative involving Linköping University, Karolinska Institute, Stockholm University, and Mid Sweden University focusing on digital mental health solutions for refugees and migrants. We conducted a concurrent mixed methods study with a quantitative and a qualitative part, recruiting participants and collecting data from June to October 2022. All participants completed the procedures for both parts, however, qualitative and quantitative data were analyzed and reported separately. Ethical approval was granted by the Swedish Ethical Review Authority (2020-00214).

### Recruitment and participants

We recruited 71 participants on site in several non-clinical settings (at an asylum housing facility, adult education classes for immigrants, non-governmental organizations and language cafés) with the help of local personnel, community members and interpreters. Interested persons could approach us directly, contact us later independently or through local collaborators. The inclusion criteria were having a refugee background, literacy in Arabic, Farsi, Dari, Swedish, or English, minimum age of 18, and currently living in Sweden. Refugee background was self-defined by the participants according to the UNHCR definition of having crossed national borders due to war, conflict, persecution, threats, or similar reasons, seeking protection in another country (here Sweden) (United Nations High Commissioner For Refugees, 2010). To ensure eligibility, the refugee background criteria were explained during recruitment and confirmed prior to commencing the study procedure. Emphasizing the refugee experience, we included participants irrespective of their legal refugee- or residence status. The sample size was calculated for the psychometric validation of the i-TAP, please see Meurling et al. (2026).

We interviewed 32 females and 39 males, between the ages of 19 and 69 (*M* = 39.2, SD = 13.1). The participants had been in Sweden for up to 9 years, and 19 (26.8%) had no residence permit, 18 (25.3%) had temporary residence permit, and 34 (47.9%) had permanent residence permit. Participants completed the interview in their preferred language, resulting in 31 interviews in Arabic, 14 in Dari, 15 in Farsi and 11 in Swedish. Interpreters were used in 28 (47%) of the non-Swedish interviews, the remaining were conducted by bilingual psychologists. See Table 1 for demographic characteristics of the sample.

**TABLE 1:** Demographic characteristics of the sample (*N* = 71).

Characteristic	Frequency	Percent
Nationality		
Syria	25	35.2
Afghanistan	19	26.8
Iran	13	18.3
Palestine	5	7.0
Other (*n* < 5)*	9	12.7
Sex		
Female	32	45.1
Male	39	54.9
Time in Sweden		
<1 year	10	14.1
1–3 years	17	23.9
4–6 years	19	26.8
7–9 years	25	35.2
Education		
University MA	10	14.1
University BA	10	14.1
High school	26	36.6
Primary school	19	26.8
Vocational qualification or other	6	8.5

*Iraq, Eritrea, Tajikistan, Somalia, Sudan, Kurdistan

### Procedure

As described, the interview data for this study were collected as part of a larger data-collection. After providing digital informed consent, including information on interpreter confidentiality, participants were engaged in a three-step procedure, comprising (1) a digital survey with background questions and completion of the i-TAP, (2) a semi-structured interview about their experiences of completing the i-TAP, and (3) a structured clinical assessment. Before commencement of step 2, an additional oral consent was obtained for the interview recording. Data for the current study were gathered in step 2.

Participation took place in a private space, and interviews were held in the participants’ preferred language. The full study procedure, including the interviews, were led by persons trained in clinical psychology and assessment, henceforward referred to as psychologists, with or without authorized telephone interpreters. All psychologists received training in the study procedure. It was the same psychologist who administered all three steps in the study procedure for the same participant. During the interview and the clinical assessment, the psychologists were blinded to the results of the i-TAP. The interview recordings could not be linked to other data collected in the study.

The interviews lasted between 01.55 and 17.27 min (*M* = 7.02 min). Since the interviews were part of a longer study protocol, we had to ensure that sufficient time was allocated for the subsequent procedure, which at times resulted in very short interviews. The use of interpreters also affected the length of the interviews.

The psychologists, the main author included, have formal training and practical experience of clinical psychology and psychiatric assessment. Five of the psychologists were bilingual, two speaking Arabic, two Farsi and one Dari, all were fluent in Swedish. The bilingual psychologists had both trained and worked clinically in and outside of Sweden, namely in Afghanistan, Iran, Egypt and the UK, and had personal migration or refugee experiences. The six Swedish speaking psychologists, the first author included, had their training and work experience in Sweden.

The professional interpreters were hired through established interpreter agencies. Both male and female interpreters were engaged, all were authorized in accordance with Swedish practice (Kammarkollegiet), and some held additional authorization as healthcare interpreters. Interpreters in Sweden are bound by professional confidentiality under law.

All participants received a small reimbursement in the form of a 99 SEK supermarket gift card.

#### Topics of the interview

In the interviews, the psychologist used an interview guide with six open questions centered around participants’ experiences of completing the i-TAP and addressing mental health in a digital survey format. We formulated questions to capture usability, key dimensions of content validity and acceptability of the i-TAP, including ease of use, language, and emotional and cognitive responses. To address prospective acceptability, defined as willingness of future engagement and perceived appropriateness, we also included a question on prospective use of the i-TAP in a health care setting.

### Data analysis

Interviews were analysed using inductive reflexive thematic analysis (RTA) from a critical realist perspective (Braun and Clarke, 2022a,b). The choice of RTA was based on its emphasis on active interpretation and view of the researchers’ subjective experiences, values and knowledge as resources in analysis (Braun and Clarke, 2022b). This allowed us to analyze these data where they are situated, in conjunction with a quantitative study, and to incorporate understandings and experiences from the larger project in the analysis. RTA also requires reflection around the researchers’ influence on analysis (Braun and Clarke, 2022b), reflexivity was promoted by continuous journaling and discussions.

As researchers we share a background in clinical psychology and refugee mental health. All authors involved in the analysis also conducted interviews with the participants, and were thus very familiar with the study population, the data, and the study concept. Two of the authors are bilingual first generation migrants, and know Arabic-, and Farsi- speaking cultures well – both personally and professionally, thus contributing with linguistic as well as cultural expertise and sensitivity.

We followed the six-step RTA process suggested by Braun and Clarke (2022b). The recorded interviews were transcribed verbatim and translated to Swedish by professional translators. Translations were verified by the bilingual members of the research group. The transcripts were organized by language or interpreter-supported interviews to facilitate detection of any problems related to translations, apart from this the data set was analyzed in its entirety. The first author conducted all stages of analysis. Familiarization involved organizing data and reading the transcripts, writing down reflections and impressions. The first author coded the data inductively and semantically. Codes were continuously refined. Initial themes were discussed with the last author (ER), who brought new perspectives. Themes were generated and refined using mind mapping techniques, including rounds of going back to codes and transcripts. To bring in additional perspectives to the analysis, we invited the third (YD) and fourth author (AG) who had highly relevant cultural and linguistic knowledge of the study population. Themes were discussed in the team of four authors to combine perspectives. Final themes were developed and participant quotes translated to English by the first author. The report has been reported in accordance with The Big Q Qualitative Reporting Guidelines (BQQRG) by Braun and Clarke (2025). For an illustrative example of the progression from codes to themes, see Supplementary Additional File 1.

## Results

**FIGURE 1:**
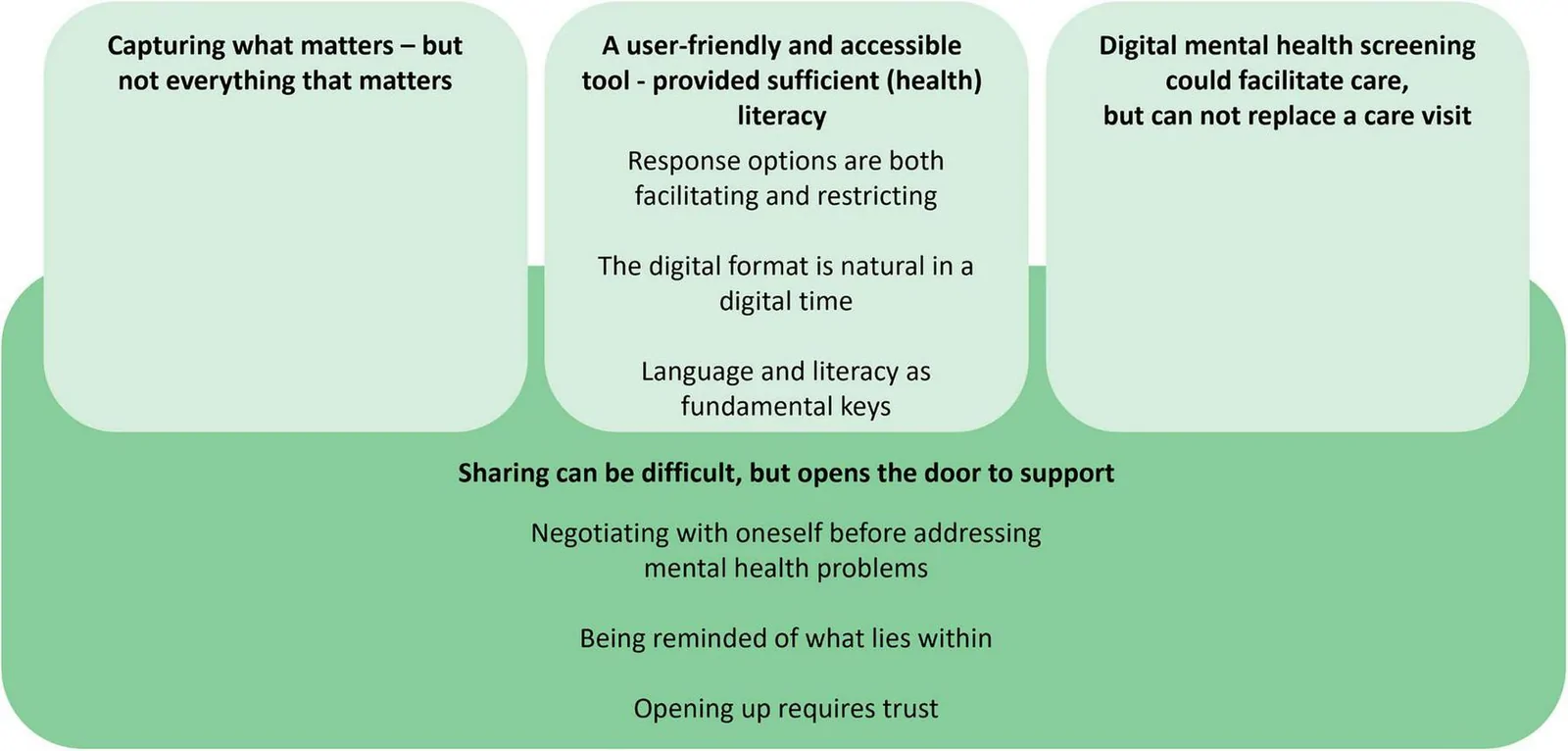
Overview of themes and subthemes.

The analysis resulted in four themes and six subthemes. The first two themes “*Capturing what matters – but not everything that matters*” and “*A user-friendly and accessible tool - provided sufficient (health) literacy*” highlight the content and usability of the i-TAP. The third theme, “*Digital mental health screening could facilitate care, but can not replace a care visit*,” centers around issues relating to prospective implementation. In the fourth theme, “*Sharing can be difficult, but opens the door to support*,” emotional, cultural and attitudinal aspects of addressing mental health issues in general, and with a digital screener, are put forward. The fourth theme can be understood as an interpretative backdrop to the other three themes, and highlights issues central to the overall acceptability of the i-TAP. Please see Figure 1 for an overview of themes and subthemes.

### Capturing what matters–but not everything that matters

The questions in the i-TAP were perceived as relevant and important. Many participants recognized themselves in the described symptoms and said that the questions captured difficulties and suffering common among refugees.

*The questions were meant for people who feel under pressure, particularly for us who have applied for asylum, waited, and then received a rejection. The survey covered a lot, it was a good experience. (12, Arabic)*

*I started the survey and felt that it was about me. The problems it brought up are similar to what I deal with everyday, so it was easy to answer. (6, Arabic)*

Sleep was the one specific topic most often reported as important by the participants. This was motivated by the strong relation between sleep, health, and daily functioning, as well as acknowledgment of how sleep is negatively affected by difficult experiences. Participants also appreciated that the i-TAP addressed pain, fear, tiredness, anger, stress, worry, depression, restlessness, and physical symptoms related to psychological problems.

*Sleep, sleep is so important. People say that if you don’t sleep well, life just doesn’t work. Your brain can’t keep up. Sleep is fundamental. (18, Dari)*

Many pointed out that refugees often have experienced multiple adverse events, and that this affects their mental health. Participants therefore valued the inclusion of questions about traumatic events and difficulties stemming from such experiences: *…they were good questions. They were questions that we have in our lives. These are terrible hardships, very difficult experiences that we have been through. (19, Dari)*

However, the participants also expressed a wish to elaborate more on their experiences. Many asked for more questions about their personal history and current situation, emphasizing that each story is unique and that the survey did not capture the full extent of what refugees may experience. For example, they asked for a more thorough elaboration on significant and potentially traumatic events, as many struggled with more recent or ongoing hardships.

*The survey is about my life, but not about everything in life. (70, Farsi)*

*It would be good if you asked more about our background, because we’re all different. One person might’ve had a great life back there and come to a place that feels good, while another might’ve had it bad there and bad here, and for that person things just get even worse. (53, Swedish).*

Several participants suggested that the specific questions about mental health should be complemented with questions regarding daily life, relationships and family life. Furthermore, various aspects of integration, including one’s role in and relation to society, were pointed out as central to mental health. The reciprocal relationship between the integration process and mental health was emphasized, with examples of loneliness and struggles to work or study. Against this, there was a request for questions regarding income, work, discrimination, the asylum process, and received social and structural support in Sweden.

*So, in the survey, the focus was only on the individual, not, for example, um, the relationship between the person and others. That should probably be included as well. (50, Arabic)*

*You know, work makes up 70% of your mental well-being. If you don’t have a job, of course you have problems. There is nothing about that in the survey. Income and stability are very important in a developed country like Sweden. (5, Arabic)*

Furthermore, participants highlighted that personal history and current circumstances can shape how one responds to the questions, which in turn affects how their answers should be interpreted and understood. In this regard, the participants expressed a will to explain and nuance their answers, an option not available in the i-TAP.

*Well for example, about the sleep problems that I have, we have different reasons. It could be that I might be nervous and just sleep badly. But it could also be that I have a little girl who wakes me up several times during the night. (34, Farsi)*

Finally, several participants noted the absence of questions addressing positive aspects of mental health in the i-TAP. The participants requested topics such as well-being, future plans, and positively framed questions to complement the i-TAP.

*In this survey, the focus was on negative feelings. It could have included positive feelings as well. There could have been questions about how you felt when arriving here, how happy you were, how your life has improved…but it was mostly about…something negative. (31, Farsi).*

### A user-friendly and accessible tool - provided sufficient (health) literacy

Variations of *It was good, it was easy (24)*, were two of the most common statements about completing the i-TAP. Overall, the participants felt that the survey was user-friendly and easy to complete, although a few found it lengthy. Participants appreciated being able to read and answer the questions at their own pace, as the questions required reflection. This underscored the need for ample time to complete the i-TAP.

*They (the questions) are easy to understand and easy to answer, no problem for me. (66, Arabic)*

*I think it’s better to, um, read and answer, rather than just being asked a question and having to respond right away. It gives you some time to think. You can understand the question better, and then give your answer. (53, Swedish)*

#### Response options are both facilitating and restricting

The survey format with response options was familiar to most participants; however, a few participants asked for more explicit instructions. Two participants observed the inconsistency in response options and time span of the questions and found this confusing. The explanations and examples given throughout the i-TAP were perceived to facilitate completion, and it was suggested that these could be complemented with reminders of the general topic of the questions at hand, e.g., “sleep problems.”

*I had no difficulties with the questions, but sometimes it was hard to understand how to choose between the answers. (18, Dari).*

The predefined response format was seen as both reassuring and limiting. Some participants found the response options clear and comprehensive, while others wished for more choices to allow for greater nuance. In line with many participants’ wish to tell more, free-text responses were suggested to allow more detailed and precise answers, enabling users to express themselves in their own words. However, others saw drawbacks with this, noting that free-text responses would be more demanding, risk omissions, or could be misunderstood. One participant proposed adding optional comment fields alongside the questions to allow users to elaborate, explain, or pose questions if they wished.

*That true feeling inside of you might not come through just by picking between the given answers. I tried, as best as I could…what you wanted… to pick the answer closest to my inner feelings…overall, it went well. (27, Farsi)*

*It was actually really good, you don’t need to explain more than what’s already there. It was good. (71, Arabic)*

#### The digital format is natural in a digital time

Although some participants reported a preference for answering surveys with pen and paper rather than digitally, many found both formats acceptable, and most favored the digital format if given a choice. The digital format was largely perceived as easy, convenient and efficient, and many found it familiar and natural, some referred to the digital era in the world: …*Answering online is easy, we live in an internet world. (14, Arabic)*

*It was easy. Typing online is faster than writing by hand and handing in papers, online is quick and easy. (35, Arabic).*

#### Language and literacy as fundamental keys

Regardless of the language used in the i-TAP, some participants expressed difficulties understanding some of the questions, while the majority found the language easy to understand and culturally appropriate. Dialectal differences and reading difficulties were mentioned as aggravating factors, and access to language support as a facilitating factor for comprehension and completion of the i-TAP.

*The questions are very clear. And it’s exactly how I would put them if I were the one asking – I’d ask them this way (71, Arabic)*

Several participants acknowledged that a digital survey (like the i-TAP) requires reading proficiency and that one’s educational background affects how easily one can understand and respond to questions about mental health. Some participants reported being more comfortable or literate in Swedish, while others appreciated the possibility to shift between languages in the i-TAP. One participant noted that individuals who fled their home at a young age, may have limited proficiency in both first and second languages, potentially hindering comprehension despite good literacy.

*Because I haven’t gone to school and haven’t studied, I found the questions that require that you’ve gone to school a bit hard to understand. (48, Dari)*

*I know Arabic, I can read Arabic fluently, but for these questions I found it to be easier in Swedish. (61, Swedish)*

Lastly, many participants emphasized that the option of responding in one’s preferred language was a prerequisite to be able to complete the i-TAP, highlighting the importance of language availability.

*It could have been difficult if I had to answer in Persian (Farsi) – I don’t speak Persian, I speak Dari. (44, Dari).*

*The language is the key to every room, the key to everything. (31, Farsi)*

### Digital mental health screening could facilitate care, but can not replace a care visit

The majority of the participants were positive about the prospective idea of completing the i-TAP in a healthcare setting, expressing it as acceptable, valuable or beneficial. One commonly mentioned benefit was that it could give healthcare professionals information about the patient’s mental health prior to the appointment, potentially saving time and improving the quality of the consultation.

*It would make things easier for me and the doctor during the time we have together. Because when I have answered, they kind of know how I’m doing. Then I don’t have to say or explain as much. (42, Swedish)*

*Of course, I don’t mind. It’s no problem. I think it’s normal to answer everything, whether its online or on paper, because you need to, you need to answer the questions since it concerns our mental health. (64, Arabic)*

The digital format was largely seen as positive and practical, with time efficiency and flexibility being key advantages. The freedom to complete the survey at a preferred time and place, such as in the comfort of one’s home, was associated with increased privacy and opportunities for reflection. Many regarded a digital questionnaire as a suitable way to address mental health; a few even noting that answering the same questions in an interview could feel stressful.

*It’s the modern way of doing things. You can communicate no matter where you are, at the asylum housing, at home…you don’t have to go to the health clinic. (4, Arabic)*

*I think it was easy, easier than talking. It was smooth, really. Yeah, I think this is easier than answering someone in person. Just tapping on the iPad is easier. (51, Swedish)*

In contrast, the same participant also reflected on when a digital questionnaire is not suitable “If someone is really ill and in need of care, they shouldn’t have to deal with an iPad or computer, they should rather get help from a person (51).” Along the same line, some emphasized the importance of meeting face-to-face in situations of distress, as it offers a sense of security not provided by a questionnaire. Moreover, other participants deemed a digital questionnaire an unsuitable approach and preferred to see a health care professional for a mental health assessment. Reluctance was expressed toward completing the i-TAP in a health care setting, and they emphasized that the i-TAP cannot replace a healthcare visit. In addition, they argued that a survey with pre-defined response options is restricting and simplifying and cannot fully depict one’s mental health.

*It feels a bit dry, like I can’t really convey the full picture of what I am feeling inside. There has to be some kind of interaction. (2, Arabic).*

Although some participants stated preferring personal contact, many still found the i-TAP acceptable, seeing how the two forms of assessment could complement each other. If implementing the i-TAP, participants emphasized the importance of receiving an introduction to the procedure, with information and instructions available in one’s first language. Meeting healthcare staff prior to administration was also suggested. Furthermore, several participants underlined that a key condition for implementing the i-TAP in healthcare would be that the results are received by someone, lead to meaningful action, and are accompanied by available support. It should not be a matter of asking questions for the questions own sake. Embedding the i-TAP in a clear context could thus be considered a prerequisite for participants’ willingness to engage with the i-TAP in healthcare settings.

*I want to share so I can get help. If I am opening up to someone who is only listening, I won’t gain anything from it. (3, Arabic)*

*It can be hard for the person to respond if it’s just a list of questions to be compiled, without any contact with them and if you’re not familiar with their problems, if it’s just about questions and answers. (25, Farsi)*

### Sharing can be difficult, but opens the door to support

Many participants emphasized the importance of addressing mental health, even though it can be difficult. Several described mental suffering as something private or sensitive, and one participant associated it with shame. Others found it easy to speak about mental health, and several expressed that they answered the i-TAP sincerely, indicating acceptability of the topic.

*Of course, it’s (mental health) one hundred percent private, but at the same time, if someone asks, it’s good to answer. I thought these questions were good to answer. (38, Dari)*

#### Negotiating with oneself before addressing mental health problems

*You know, unfortunately these cultural and individual tensions make you constantly battle yourself. But then you come to a point where you start considering asking for help. (25, Farsi)*

Participants reflected on cultural differences between Sweden and their countries of origin. Several noted that in Sweden, discussing mental health is perceived as safe and normalized, and some concluded that stigma in their home country does not necessarily translate to stigma in Sweden. For some, answering questions about mental health was a completely new, yet acceptable, experience in the current Swedish context. However, fear of stigma and potential negative consequences of sharing mental health issues was also expressed. Thus, participants expressed both normalizing and hesitant positions to sharing mental health issues. This duality was reflected in considerations made based on awareness of cultural differences, for example in emotional expression, and how cultural barriers may need to be navigated in order to seek care. This cultural awareness and contrast was also reflected in comments normalizing mental health, spanning from the right to access mental health services, to the idea that mental health is okay to talk about, to explicit rejections of stigmatizing beliefs.

*I don’t think it’s particularly private, a sick person needs to get help from a doctor. It’s just like if we had heart problems or needed dental care. You also need to do psychological check-ups. (31, Farsi)*

*To be honest, I’ve never filled out a questionnaire about my mental health before. If I had told someone in (homecountry) that I was feeling tired, they would’ve called me crazy and taken me to a mental hospital. No one ever asks about your feelings there, not doctors, not family, no one. So this was the first time I did something like this. (1, Arabic)*

#### Being reminded of what lies within

*The questions about how it feels inside make you aware of how it actually feels inside. (42, Swedish).*

Addressing mental health through the i-TAP evoked emotional reactions and reflections among some of the participants, and as noted by the participant above, the questions naturally lead one to reflect inward. Feelings of sadness, hopelessness and concerns about the future were described by some participants. Others reported that the questions revived memories – often, though not always, of difficult and painful events, which was strenuous. These recollections evoked grief, fear, and anxiety, which at times also was manifested during the interviews.

*It went well, it’s just that I felt my heart clench in my chest – as if my heart had grown heavy. (23, Farsi).*

*It’s a bit mixed you know. Even if it was interesting to answer these questions, you feel tired from it, because some questions remind you of things, um, you’d rather not remember again. (50, Arabic)*

For several participants, completing the i-TAP was an emotionally positive experience, often related to feeling acknowledged and listened to. Others experienced completing the i-TAP as emotionally difficult but also meaningful, or expressed that it made them aware of struggles as well as strengths. All participants who reported emotional reactions, whether negative or positive, were open to completing the i-TAP again and expressed support for its use in clinical settings.

*Questions about positive and negative thoughts during these two weeks. That was very important. When I talked about these two weeks…I felt a deep sense of calm. Someone is listening to me. (28, Farsi)*

*Yes, because there are things in life that we carry with us–like a backpack we’re unaware of–and when a question like this comes up, something clicks. ‘Oh my God, why have I been carrying this around?’ It makes you realize it’s unnecessary. (70, Farsi)*

#### Opening up requires trust

While acknowledging that opening up about mental health can be difficult, many participants stressed the importance of doing so – noting that talking about it may bring relief, and foremost, how confiding in someone is necessary to get help and support. However, the participants made it clear that this requires trust. Feeling safe and understood, with the person and in the context, was a precondition to discuss mental health. Several participants expressed trust in health care professionals, with confidentiality and professional training being important aspects, while others expressed distrust and worry that mental health information could lead to negative consequences if accessed by other authorities.

*If you trust the other person, you can bring things up and feel a bit relieved. You can feel that relief while they can also help you. But if you don’t know them and you’re afraid…then I don’t share. (29, Farsi)*

*Well, it depends. If you talk to a doctor or other professionals, then yeah, you can talk about it a bit. But with regular people, not really, no. (49, Farsi)*

Many participants also put forward the importance of seeking and receiving care for mental health issues, and to ask refugees about their mental health, displaying knowledge and belief in health care and mental health interventions.

*I think it is good (to address mental health), because when you’re new in this country, and you’re not feeling well, then, you need someone to talk to, like a psychologist or psychiatrist or someone who can help. (47, Dari)*

In summary, reflecting the various aspects in this theme, the idea of healthcare inquiring about mental health, through the i-TAP, was perceived as positive and important, and as a potential first step toward support.

## Discussion

This study investigated the experience of completing an online mental health screening procedure, the i-TAP, among refugees in Sweden. Four themes were generated, encompassing the i-TAP itself, its potential use in health care, and emotional and cultural aspects of mental health screening and help seeking: *Capturing what matters – but not everything that matters*, *A user-friendly and accessible tool - provided sufficient (health) literacy*, *Digital mental health screening could facilitate care, but can not replace a care visit*, and *Sharing can be difficult, but opens the door to support.*” Participants were mainly positive to the i-TAP, its use in health care and to address mental health in general. However, they raised concerns about trust, accessibility, and the importance of follow-up of a mental health screener.

To contribute to the validation of the i-TAP among individuals with a refugee background, a specific focus of this study was to investigate the acceptability, content validity, and usability of the i-TAP from a user perspective. The results will be discussed in relation to these concepts, as well as potential improvements and clinical implications.

### Acceptability

The findings indicate high overall concurrent acceptability, as the participants were largely positive toward the i-TAP and found it important and relevant. Interestingly, the emotional discomfort the i-TAP evoked for some participants did not contraindicate mental health assessment but was rather an expected and accepted part of the process. These findings on emotional reactions show how even a self-assessment survey can raise meaningful reflection.

The navigation of culture and stigma in addressing mental health was another aspect to consider, as the Swedish context seemed to increase acceptability of the i-TAP, but it was also negotiated in relation to participants’ heritage culture and explanatory models of mental illness. This contextualized nature of acceptability is likely sensitive to acculturation and can be expected to vary in the population and over time. Moreover, concurrent acceptability was not found among all participants, indicating that alternative ways of mental health assessment should be available.

Our findings show that prospective acceptability, including the perceived appropriateness of the i-TAP and the expressed willingness to use it in a health care setting, came with two conditions: follow-up and trust. The i-TAP was deemed acceptable if implemented with rationale and follow-up in a clear and trusted context. With these prerequisites fulfilled, the willingness, or intention to use the i-TAP was high. As authors, we share our participants’ views on these conditions for implementation. A blended approach is also supported by the literature, integrating digital interventions with - not in replacement of – real-world clinical settings, is proposed to optimize the benefits of digital health tools (Ng et al., 2025; Smith et al., 2023). Furthermore, participants emphasized that the i-TAP should not replace a care visit, which is in line with the intention of the i-TAP to complement routine care, which requires trust and presupposes that results are followed up and acted upon.

Trust has been found to be of particular importance to individuals with a refugee background, given uncertain, unsafe and adverse refugee trajectories (Essex et al., 2022; Shannon et al., 2016). As trust is relationally, contextually and temporally dependent, it often needs to be restored in resettlement (Essex et al., 2022). Consequently, establishing trust is a significant component for successful mental health referrals among resettled refugee populations (Shannon et al., 2016). Although the participants in this study expressed a general trust in health care professionals, settings and interventions, trust should never be assumed and always carefully considered in every health care setting.

The digital format was found to contribute to the willingness to use the i-TAP by enabling availability, flexibility and efficiency. For most participants, the digital format was a facilitating aspect related to usability, time required, and convenience, in line with previous studies (Whitehead et al., 2023). These qualities could increase accessibility by bridging practical barriers to care, such as transport, finances, and time constraints, previously reported by refugees (Byrow et al., 2020; Mabil-Atem et al., 2024). Important to note is that this sample was generally familiar with the digital format and that no concerns about access to internet or devices were reported, which likely contributed to the positive views of a digital tool. Furthermore, in contrast to previous findings participants were not concerned about privacy (Mabil-Atem et al., 2024), which could be understood as part of the expressed trust in health care services, but also a potential effect of self-selection bias. The opportunities to complete the survey in one’s preferred language and at a convenient time, are other bridges facilitated by a digital format. Furthermore, the participants identified several benefits of completing a digital survey prior to a health care visit, demonstrating the view that asking about mental health is important, further supporting the prospective acceptability of the i-TAP as part of a blended approach in care services.

To conclude, although some did not support the idea of a digital mental health screener, the i-TAP was overall found to be an acceptable and accessible way to address mental health problems, a way to initiate conversation on the topic, and to identify individuals with mental health problems - if it is implemented and followed-up in a trusted context.

### Content validity

The participants found the content of the i-TAP highly relevant and suitable for individuals with a refugee background. Sleep problems and difficulties related to traumatic experiences stood out as particularly important topics to address, potentially mirroring the high prevalence of these problems in this sample (Meurling et al., 2026). It is also possible that sleep problems and more collective traumatic experiences and related difficulties are easier to name, less stigmatized, and well-known due to being widely shared among individuals with a refugee background (Hassan et al., 2015; Blackmore et al., 2020; Lies et al., 2019).

Regarding comprehensibility, the questions were largely perceived as easy and straightforward, the language accessible, and the digital format easy to use, indicating high comprehensibility and usability of the i-TAP in the languages included in this sample. This aligns well with our previous results where a vast majority of the same participants rated the i-TAP as easy to understand and complete (Meurling et al., 2026). Furthermore, language itself was key to accessing the i-TAP at all, not only its comprehensibility, underscoring its role as both a facilitator and barrier (Mabil-Atem et al., 2024; Whitehead et al., 2023). However, language proficiency varied, and it is important to acknowledge that difficulties also emerged. Our findings indicate that comprehensibility was not only dependent on reading skills; education, including general language proficiency, formal education, and mental health literacy also facilitated understanding - all ongoing processes for individuals who are resettling in a new country and culture. The fact that some participants chose to participate in Swedish or made use of the possibility to switch between languages, could be understood as examples of these processes. Other possible explanations behind this choice include that responding in a second language is less emotion evoking (Harris, 2004), that mental health literacy and vocabulary may be more closely tied to the Swedish context, a reluctance to use telephone interpreters, or simply that their first language was not provided as option in the study. In summary, it is essential to give the opportunity to choose one’s preferred language when addressing mental health, and to not take comprehension of written material for granted.

Concerning comprehensiveness, our results show that on the one hand, the i-TAP was found to be extensive and comprehensive, and on the other, there was a clear wish and need to tell more which highlights the inherent limitations of a screening tool. In line with previous findings (Behrendt et al., 2023; Farahani et al., 2021), the participants related mental health both to traumatic or difficult experiences and to factors in their ongoing daily lives. These findings point to the importance of addressing postmigration factors such as social bonds, employment, discrimination, and integration in relation to mental health, whether it be in a screening or clinical assessment. For this population, it is also important to explore potential occupational or socioeconomic downgrade, as well as clashes between more collectivistic and individualistic social values (Herold et al., 2024). Additionally, the wish to say more included positive dimensions of mental health. In the i-TAP, the most positive alternative and result is absence of symptoms. This could be criticized against the WHO definition of health as well-being, not the absence of disease (World Health Organization, 1948) and comprehensiveness. However, comprehensiveness must be weighed against feasibility, as there is a cost to adding items. The i-TAP partially overcomes this dilemma as the tiered design reduces the individual item burden with retained accuracy (Meurling et al., 2023, 2026). Ultimately, the purpose of the i-TAP is to identify mental health problems, and thus, it is neither a comprehensive nor holistic screener for mental health.

In summary, it could be argued that the i-TAP was perceived as relevant, comprehensible, and had acceptable comprehensiveness as a screener, while bearing in mind that screening for specific symptoms is not enough to fully assess mental health.

### Further development

To meet the wish to express more nuance, details or context, adding the possibility to leave free-text answers would be a feasible modification. This would also enable users to add issues not addressed in the i-TAP, which could increase comprehensiveness for the individual and be of great value to the further assessment. Another alternative would be to add questions or scales addressing postmigration factors, with the purpose to get a broader picture of personal circumstances and contextualize potential mental health problems. However, even if adding such questions would increase the perceived comprehensiveness of the i-TAP, it would alter its purpose. Therefore, and against our results, we suggest that these issues are addressed either in addition to the i-TAP or in a follow-up assessment, rather than including them as part of the i-TAP itself.

The usability of the i-TAP could be improved by adding recurring instructions and guidance throughout the survey. This would be of particular importance for those answering on a smartphone, since less content is visible on smaller screens. This could also help reduce confusion over varying response options and time spans. Making these uniform was not considered as the i-TAP was built with validated scales. Nevertheless, evaluating new and uniform time spans and response options could be of interest in future work and enhance the usability of the i-TAP.

Regarding language, we propose including a recurring item about comprehension of the questions, to enable detection of difficulties that could be important to the interpretation of the results. Integrating a function to have the questions read aloud would be a feasible way to address literacy difficulties. However, both suggestions should serve a supportive function. In cases of low literacy, other options should be offered.

### Clinical implications

The results of this study indicate that the i-TAP is an acceptable and feasible first screener that could be implemented in health care settings to briefly assess mental health problems among individuals with a refugee background. For implementation to be successful, our results point out the importance of establishing trust, offering a clear rationale with instructions, and scheduled follow-up, all in the respondent’s preferred language.

In a clinical setting, it is important to acknowledge the limitations of a self-assessment survey, such as the i-TAP. Its purpose is not to offer a full mental health screening or clinical assessment, but to screen for common mental health problems among individuals with a refugee background. As such, it should be complemented by a clinical assessment when indicated. Our findings further suggest that health care professionals should address current stressors, including post-migration factors such as discrimination and acculturation, personal history, positive aspects in life and well-being. Furthermore, it is important to be sensitive to potential ongoing inner cultural negotiations, for example by giving patients time, make room for questions, and address the topic on several occasions.

Given the central role of language shown in the findings, it is important to address language proficiency and give individuals the opportunity to continue the conversation in their preferred language. If respondents are hesitant to answer a digital screener, investigate digital literacy and acceptability. Alternatives such as paper surveys, language support and in-person meetings should be considered and offered.

### Strengths and limitations

From the larger perspective of validating a screening instrument, it is important to address limitations in relation to transferability and the convenience sampling used. There is a risk that the perspectives and experiences presented by the participants are positively biased, as participation was self-selected, and that the acceptability of the i-TAP is lower in the general refugee population. Furthermore, communication of critical remarks or negative experiences might have been impeded by courtesy bias, power imbalance, and politeness as the interviews were conducted by psychologists representing the i-TAP. As the bilingual psychologists in the research group have highlighted, politeness and respect are likely strong cultural values among our participants. Moreover, the authors’ preconceptions, beliefs, and wishes might also contribute to the results (Braun and Clarke, 2024). The themes we produced, along with the predominantly positive perspectives found, might reflect an unintended emphasis on outcomes consistent with the long-term objectives of the research project. According to RTA, this is inevitable and pre-understanding is part of analysis, which therefore warrants reflexivity (Braun and Clarke, 2024). Thus, to address these possible threats and increase fidelity, yet without aiming to withdraw ourselves from the analysis, we actively reflected on these issues and were careful to acknowledge negative comments throughout the analysis. Against this, and with the cultural perspectives represented among the authors, we believe that the familiarity with the participants, the population, and the larger project contributed to a more nuanced and contextualized understanding of the participants’ statements.

The credibility and transferability of the findings also depend on the data quality and information power (Malterud et al., 2016) in this study. The interviews were very brief, resulting in rather thin data from each participant, which is a weakness. Relatedly, the use of telephone interpreters may have affected communication and data quality. Although interpretation quality was generally deemed good, some participants may have shortened their answers, either from frustration or to adapt to the interpreted format, while for others the same format provided additional time to reflect. Nonetheless, we strongly believe that a brief interview, with or without interpreter assistance, allowed for more nuanced data than would have been obtained through additional free-text survey questions about participants’ experiences. In addition, the sample was large, with few inclusion criteria, which increases the chance of variation in the experiences investigated and the utility of data (Levitt et al., 2017). We further argue that having several different interviewers is a strength, as it also contributes to increased variation. Thus, even though the individual interviews were short and potentially influenced by the use of interpreters, we consider the data to be of adequate quality and sufficient information richness for the purposes of this study. Lastly, we acknowledge that the sampling, interviews, and analysis of this study were conducted within a quantitatively framed project. While purposeful sampling and longer interviews could have increased richness and resulted in a deeper understanding, the current format served its purpose to collect the experiences from all participants in the study.

### Conclusions and implications

In our previous psychometric validations, we have shown that the i-TAP is an accurate and efficient screening tool for identification of clinically relevant symptoms of depression, anxiety, PTSD, and insomnia among individuals with a refugee background in Sweden. With this study, we can further conclude that it is also perceived as acceptable by the target population, with the potential to facilitate assessment and access to services. Based on the findings in this study, we have also identified some developments that could further improve the usability of the i-TAP. The next step toward implementation would be to address acceptability and readiness among health-care providers. In addition, investigating how trust can be established and kept in a mental health care setting would be of great interest and importance.

## Data Availability

The datasets presented in this article are not readily available because of ethical restrictions protecting participant confidentiality. Requests to access the datasets should be directed to jennifer.meurling@miun.se.
