# Supplementary material for: “It’s the modern way of doing things”: a qualitative study of user-experience, acceptability, and content validity of a digital mental health screener for refugees
Source: Front Psychol. 2026 Sep 11;17:1902434. doi: 10.3389/fpsyg.2026.1902434 (PMC13612513; doi:10.3389/fpsyg.2026.1902434)
Supplement: Supplementary file 1 [file Table_1.docx]

**Additional file 1**. Illustration of the analysis

Table 1 illustrates the analytical process and the progression from codes to final themes. This was an iterative, recursive rather than linear process, and this table offers a simplified overview. In practice, initial, candidate, and final themes were repeatedly refined against the codes and transcripts.

Table 1. Example of the progression from code to final theme

| **Code** | **Initial theme** | **Candidate theme** | **Final theme** |
| --- | --- | --- | --- |
| Recognising oneself in the symptoms | Relevant content | The survey is about my life | Capturing what matters – but not everything that matters |
| The survey asks questions about refugees' everyday life and situation | Relevant content |  |  |
| Lacking questions about the underlying reasons behind the answers to the questions | Missing aspects | A desire to share more |  |
